# Neonatal mortality in two districts in Indonesia: Findings from Neonatal Verbal and Social Autopsy (VASA)

**DOI:** 10.1371/journal.pone.0265032

**Published:** 2022-03-14

**Authors:** Poppy E. Deviany, Philip W. Setel, Henry D. Kalter, Trisari Anggondowati, Martini Martini, Fitri Nandiaty, Kamaluddin Latief, Emily H. Weaver, Tika Rianty, Anhari Achadi, Sri Wahyuni, Stefania W. Setyaningtyas, Nila R. Haryana, Luna M. Mehrain, Endang L. Achadi

**Affiliations:** 1 Center for Family Welfare, Faculty of Public Health, Universitas Indonesia, Depok, Indonesia; 2 Vital Strategies, New York, NY, United States of America; 3 Institute for International Programs, Department of International Health, Johns Hopkins Bloomberg School of Public Health, Baltimore, MD, United States of America; 4 Faculty of Public Health, Universitas Diponegoro, Semarang, Indonesia; 5 Carolina Population Center, The University of North Carolina at Chapel Hill, Chapel Hill, North Carolina, United States of America; 6 Faculty of Public Health, Universitas Airlangga, Surabaya, Indonesia; 7 USAID Jalin Project, implemented by DAI Global LLC, Jakarta, Indonesia; University of Dhaka: Dhaka University, BANGLADESH

## Abstract

**Background:**

The Government of Indonesia is determined to follow global commitments to reduce the neonatal mortality rate. Yet, there is a paucity of information on contributing factors and causes of neonatal deaths, particularly at the sub-national level. This study describes care-seeking during neonates’ fatal illnesses and their causes of death.

**Methods:**

We conducted a cross-sectional community-based study to identify all neonatal deaths in Serang and Jember Districts, Indonesia. Follow-up interviews were conducted with the families of deceased neonates using an adapted verbal and social autopsy instrument. Cause of death was determined using the InSilicoVA algorithm.

**Results:**

The main causes of death of 259 neonates were prematurity (44%) and intrapartum-related events (IPRE)-mainly birth asphyxia (39%). About 83% and 74% of the 259 neonates were born and died at a health facility, respectively; 79% died within the first week after birth. Of 70 neonates whose fatal illness began at home, 59 (84%) sought care during the fatal illness. Forty-eight of those 59 neonates went to a formal care provider; 36 of those 48 neonates (75%) were moderately or severely ill when the family decided to seek care. One hundred fifteen of 189 neonates (61%) whose fatal illnesses began at health facilities were born at a hospital. Among those 115, only 24 (21%) left the hospital alive–of whom 16 (67%) were referred by the hospital.

**Conclusions:**

The high proportion of deaths due to prematurity and IPRE suggests the need for improved management of small and asphyxiated newborns. The moderate to severe condition of neonates at the time when care was sought from home highlights the importance of early illness recognition and appropriate management for sick neonates. Among deceased neonates whose fatal illness began at their delivery hospital, the high proportion of referrals may indicate issues with hospital capability, capacity, and/or cost.

## Introduction

In 2018, neonatal mortality accounted for 2.5 million deaths globally, around 47% of all under-five deaths worldwide [1]. Preventing neonatal mortality is essential to meet child survival goals. Through the Every Newborn Action Plan, governments and partners across the globe have committed to reduce the neonatal mortality rate (NMR) in all countries to 10 deaths or fewer per 1,000 live births by 2035 [2]. Indonesia has a high NMR, estimated to be 15 per 1,000 live births in 2017 –higher than many neighboring countries [3–5].

Determinants of neonatal death include not only neonates’ health conditions but also maternal complications; access to and quality of health care; and socio-economic and behavioral factors [6, 7]. Birthing at home and mothers’ lack of knowledge of danger signs for neonatal illnesses have also been shown to increase the risk of neonatal death [8]. Low maternal education and a lack of autonomy may prevent mothers from seeking health care and can influence decision-making in the family that affects children’s health [2]. Distance to a health care facility, lack of transportation, cost, trust of health care providers, and perception of the importance of traditional care are among other factors that have hindered care-seeking for neonatal complications [7, 9, 10].

The Government of Indonesia has improved access to health care services in general and especially to delivery services. The latest Indonesia Demographic and Health Survey (IDHS) shows an increase in facility-based deliveries from 56% to 74% between 2012 and 2017 [4]. In addition, implementation of the National Health Insurance (NHI) policy since 2014 has contributed to the increase in facility-based deliveries [11, 12].

Despite improvements in delivery care and insurance coverage, Indonesia’s NMR remains high. Lack of understanding about the benefits and coverage of insurance for neonates among families and health providers may contribute to barriers in seeking care for neonates’ illnesses, which then affect neonatal outcomes [13].

Monitoring neonatal mortality is complicated by issues in recording and reporting of deaths. Studies have suggested that neonatal deaths are often under-reported in routine health information systems in many developing countries [14, 15]. This issue is complicated by the possible misclassification between early neonatal deaths and stillbirths [16]. Furthermore, many developing countries have sub-optimal civil registration systems and medical certification of deaths. This leads to a scarcity of valid information on the magnitude and causes of death, particularly at the sub-national level. In addition, even with optimal civil registration, little or no information on social factors related to death is collected. This information is needed to identify potential points of intervention to reduce avoidable deaths.

The ‘Every Mother and Newborn Counts’ (EMNC) study was undertaken in Serang and Jember Districts as part of USAID’s five-year Jalin project [17] to reduce maternal and neonatal mortality in Indonesia and is intended to inform priorities and innovative intervention development. The current analysis was conducted to explore care-seeking during neonates’ fatal illnesses and identify the causes of the deaths.

## Methods

### Study design and population

This cross-sectional study examined data of all neonatal deaths from June to November 2018 in Serang District of Banten Province and from July to December 2018 in Jember District of East Java Province. These districts were selected due to the high burden of maternal and neonatal mortality and a strong commitment from the local government to address the mortality [18]. Serang District has a population of 1.5 million in 29 sub-districts and 326 villages [19] and Jember District has a population of 2.3 million in 31 sub-districts and 248 villages [20]. Most of the population in these districts resides in rural villages.

This study used modified community-based methods to identify and list all neonatal deaths in each district, namely, ‘Neonatal Deaths from Informant/Neonatal Deaths Follow on Review’ (NODE-IN/NODE-FOR) methods [21]. The original methods, ‘Maternal Deaths from Informant/ Maternal Deaths Follow on Review’ (MADE-IN/MADE-FOR), were used to identify maternal deaths [22]. In the NODE-IN/NODE-FOR methods, local informants most knowledgeable about vital events in the community were asked to list all neonatal deaths and stillbirths of which they had knowledge. They were also asked to provide information about the neonate’s age at death and contact information of the parents/family, which were later verified through interviews with the deceased’s family.

The eligibility criteria used to identify potential cases in the listing process were: 1) stillbirths and neonates who died during the six-month recall period; 2) parents of the deceased were residents of the district (proven with identification or by indicating they had been living in the study districts for at least six months prior to their child’s death).

After listing, an initial interview to confirm each case was conducted with the neonate’s mother or other caregiver who was most knowledgeable about the circumstances surrounding the fatal illness and death. Questions about signs of life, including whether the baby ever breathed, cried or moved, were asked of the respondent to verify whether the neonate was born alive or was a stillbirth. All neonates who were born alive and died on day 0–27 following birth were included in the study.

The VASA instrument consisted of the 2016 World Health Organization Verbal Autopsy (VA) instrument together with a Social Autopsy (SA) questionnaire developed by Johns Hopkins University [23]. Interviews using the VASA instrument were conducted with mothers or main caregivers of the deceased neonates. We collected information on characteristics of neonates and the parents; place of birth and birth attendants; maternal symptoms (later grouped to form maternal complications); signs and symptoms of the neonatal illness; place of death; health insurance status; and care-seeking during fatal illness. The six-month recall period was intended to minimize recall bias while still obtaining information on enough deaths for rigorous analysis.

Data collection was conducted using the Open Data Kit (ODK) platform installed on tablet computers. Data collectors were mostly graduates from midwifery or nursing academies. The data were electronically transmitted from the field to the central research office for storage and were analysed using STATA 15.0 software [24]. We conducted a descriptive analysis of the demographic characteristics of neonates and their mothers, care-seeking during neonates’ fatal illnesses, and neonatal causes of death.

The illness recognition and care-seeking processes are described based on the ‘Pathway to Survival’ framework [25, 26]. Information on signs and symptoms prior to death was used to determine the likely causes of death using the InSilicoVA computer algorithm [27, 28]. The socio-economic level of the family was measured using wealth quartiles based on a composite index of household assets (such as mobile phone, television, motorized vehicle), materials for housing construction, source of water and energy, and sanitation facilities. The wealth quartiles were derived for each district using principal components analyses [18] to assign a wealth score for each household of a death case. The households were then ordered by the score, and the score distribution was divided into quartiles, where quartile one refers to the poorest and quartile four denotes the wealthiest.

In this study, the term ‘formal care provider’ refers to a trained or certified health care provider or skilled birth attendant (SBA), regardless of whether care was provided inside or outside a health facility. An ‘informal care provider’ refers to an individual providing traditional medicines, home remedies, or non-professional treatment. Antenatal care was categorized according to the number of visits in reference to the Indonesian government’s recommended visitation schedule at the time of the study (i.e., one time during each of the first and second trimesters and twice during the third trimester–the 1-1-2 standard [4]).

Adapting the approach used by Koffi et. al. [29], neonates’ illness severity rank was determined according to feeding behavior and activity level as normal/mild, moderate, and severe. Health insurance status was categorized into 1) insured through NHI or other insurance types; 2) insured through government aid, and 3) not insured. For the second category, government aid is for those who were not covered by NHI and come from poor households; the aid can come from central and/or local government.

### Ethics

This study obtained ethical clearance from the Institutional Review Board at the Biomedical Research Alliance New York (BRANY) and at the Faculty of Public Health, Universitas Indonesia. Written informed consent was obtained from each respondent prior to participating, and each respondent was also provided a hard-copy description of the study and a consent statement with the local PI’s contact information. All the VASA study personnel were trained on ethical principles and practices for human subject research, including matters of sensitivity, confidentiality, administering informed consent, and prescribed assistance to bereaved respondents.

## Results

Nine hundred and two cases were listed by the community informants as either neonatal deaths or stillbirths. After verification with the family of the deceased, 259 (29%) of the cases were confirmed as neonatal deaths and eligible for our study. The remaining cases were ineligible mainly due to being stillbirths (31%), deaths that occurred outside the study period (22%), and gestational age less than 28 weeks (10%). A separate comparison with the district health office data found that this study captured 1.4 and 1.9 times more neonatal deaths than district records in Serang and Jember Districts, respectively.

### Characteristics of neonatal deaths and place of death

Table 1 shows that 34% of neonatal deaths occurred on the first day of life (day 0) and 79% died within the first week (day 0–6) after birth. Of all deceased neonates, 61% were male, 32% were firstborn, and 56% were from rural areas. Maternal characteristics show that 34% of mothers of deceased neonates were aged <25 years, 42% were first married when under 19 years of age, and 34% had either no education or only attended primary school. About 34% of the neonates’ mothers did not have insurance or received government aid for their health care. Among the deceased neonates seen by one or more formal care providers during their fatal illness, over half (53%) were not using any insurance or government aid when they were first seen.

**Table 1:** Demographic characteristics.

Characteristics	Serang n = 109	Jember n = 150	Total n = 259
	n (%)	n (%)	n (%)
***Neonatal characteristics***			
**Age of neonates at time of death (days)**			
0	34 (31.2)	54 (36.0)	88 (34.0)
1–6	53 (48.6)	63 (42.0)	116 (44.8)
7–13	13 (11.9)	17 (11.3)	30 (11.6)
14–20	6 (5.5)	7 (4.7)	13 (5.0)
21–27	3 (2.8)	9 (6.0)	12 (4.6)
**Gender of neonates**			
Male	66 (60.6)	91 (60.7)	157 (60.6)
Female	43 (39.4)	59 (39.3)	102 (39.4)
***Parents’ and household characteristics of the deceased neonates***			
**Maternal age (years)**			
15–19	6 (5.5)	21 (14.0)	27 (10.4)
20–24	23 (21.1)	37 (24.7)	60 (23.2)
25–29	19 (17.4)	30 (20.0)	49 (18.9)
30–34	20 (18.4)	29 (19.3)	49 (18.9)
≥ 35	41 (37.6)	33 (22.0)	74 (28.6)
**Maternal education**			
None or Primary	36 (33.0)	52 (34.7)	88 (34.0)
Junior High	38 (34.9)	48 (32.0)	86 (33.2)
Senior High	32 (29.3)	38 (25.3)	70 (27.0)
Academy/University	3 (2.8)	12 (8.0)	15 (5.8)
**Maternal age when first married (years)**			
<16	7 (6.4)	12 (8.0)	19 (7.4)
16–18	29 (26.6)	61 (40.7)	90 (34.7)
>18	71 (65.2)	74 (49.3)	145 (56.0)
Don’t know	2 (1.8)	3 (2.0)	5 (1.9)
**Parity**			
Primiparous	28 (25.7)	54 (36.0)	82 (31.7)
Multiparous	81 (74.3)	96 (64.0)	177 (68.3)
**Maternal health insurance**			
Insured	59 (54.1)	51 (34.0)	110 (42.5)
Government aid	16 (14.7)	44 (29.3)	60 (23.1)
No insurance	34 (31.2)	55 (36.7)	89 (34.4)
**Paternal education**			
None or Primary	45 (41.3)	67 (44.7)	112 (43.2)
Junior High	26 (23.9)	34 (22.6)	60 (23.2)
Senior High	34 (31.1)	39 (26.0)	73 (28.2)
Academy/University	4 (3.7)	10 (6.7)	14 (5.4)
**Residence**			
Urban	42 (38.5)	73 (48.7)	115 (44.4)
Rural	67 (61.5)	77 (51.3)	144 (55.6)
**Wealth quartile** ^			
Poorest (Q1)	24 (22.0)	48 (32.0)	
Lower middle (Q2)	35 (32.1)	33 (22.0)	
Upper middle (Q3)	24 (22.0)	34 (22.7)	
Wealthiest (Q4)	26 (23.9)	35 (23.3)	
**Use of health insurance for neonatal illness at the first formal care provider**	**n = 96**	**n = 141**	**n = 237**
Insured	33 (34.3)	36 (25.5)	69 (29.1)
Government aid	9 (9.4)	34 (24.1)	43 (18.1)
No insurance	54 (56.3)	71 (50.4)	125 (52.8)

^the index was estimated separately for each district.

All but one mother of the deceased neonates received antenatal care (n = 258), as seen in Table 2. Most mothers visited health providers for ANC (98%). However, many also visited traditional birth attendants (28%—not shown in Table 2). Of health providers, midwives were more likely to be consulted for ANC compared to doctors or nurses. Most mothers of deceased neonates (79%) received ANC in the first trimester and 89% received ANC at least four times during their pregnancy. Of those who reached term pregnancy (n = 138), 76% of the mothers had ANC visits according to the government-recommended 1-1-2 schedule.

**Table 2:** Antenatal care characteristics.

Antenatal Care	Serang n (%)	Jember n (%)	Total n (%)
**Health provider seen during ANC visits** *	**n = 108**	**n = 144**	**n = 252**
Village midwife	66 (61.1)	96 (66.7)	162 (64.3)
Primary health center *(Puskesmas)* midwife	56 (51.9)	90 (62.5)	146 (57.9)
Private midwife	62 (57.4)	78 (54.2)	140 (55.6)
General practitioner/obstetrician	35 (32.4)	64 (44.4)	99 (39.3)
Others e.g., midwife unknown, nurse, non-specified health providers	9 (8.3)	6 (4.2)	15 (6.0)
**Number of ANC visits**	**n = 108**	**n = 144**	**n = 252**
1–3	16 (14.8)	9 (6.3)	25 (9.9)
4 or more	91 (84.3)	134 (93.1)	225 (89.3)
Don’t know	1 (0.9)	1 (0.6)	2 (0.8)
**ANC by trimester**			
First trimester	81 (75.0)	118 (81.9)	199 (79.0)
Second trimester	104 (96.3)	142 (98.6)	246 (97.6)
Third trimester	88 (81.5)	133 (92.4)	221 (87.7)
**Of those who reached term pregnancy (gestational age > = 9 months)**	**n = 48**	**n = 90**	**n = 138**
Had 4 ANC visits per government schedule by trimester–sequence of 1, 1, 2	32 (66.7)	73 (81.1)	105 (76.1)

*Multiple answers are allowed.

Most neonates (83%) were born at a health facility, with nearly half (47%) born at a hospital, as seen in Table 3. Eighty-six percent of the 37 home births were not assisted by an SBA. Twenty-four percent of all deceased neonates were delivered by C-section. Nearly three quarters (74%) died at a health facility; 63% at hospital and 11% at lower level health facilities.

**Table 3:** Birth characteristics, place, and cause of death.

Characteristics	Serang n = 109	Jember n = 150	Total n = 259
	n (%)	n (%)	n (%)
**Place of birth**			
Hospital	46 (42.2)	75 (50)	121 (46.7)
Other health facility	38 (34.9)	56 (37.3)	94 (36.3)
Home^+^	21 (19.2)	16 (10.7)	37 (14.3)
On route to hospital or facility	4 (3.7)	3 (2.0)	7 (2.7)
**Birth attendants**			
Village midwife	3 (2.7)	3 (2.0)	6 (2.3)
Primary health center *(Puskesmas)* midwife	19 (17.4)	24 (16.0)	43 (16.6)
Private midwife	22 (20.2)	23 (15.3)	45 (17.4)
Unspecified midwife	18 (16.5)	16 (10.7)	34 (13.2)
General practitioner/obstetrician	26 (23.9)	59 (39.3)	85 (32.8)
Traditional birth attendant	9 (8.3)	3 (2.0)	12 (4.6)
Nurse	1 (0.9)	4 (2.7)	5 (1.9)
Others e.g., relatives/friend or non-specified birth attendant	0 (0.0)	6 (4.0)	6 (2.3)
No one	11 (10.1)	11 (7.3)	22 (8.5)
Don’t know	0 (0.0)	1 (0.7)	1 (0.4)
**Mode of delivery**			
Spontaneous vaginal delivery (without forceps or vacuum)	86 (78.9)	105 (70.0)	191 (73.7)
Assisted vaginal delivery (with forceps or vacuum)	5 (4.6)	1 (0.7)	6 (2.4)
C-section	18 (16.5)	44 (29.3)	62 (23.9)
**Place of death**			
Hospital	57 (52.3)	106 (70.7)	163 (62.9)
Other health facilities	17 (15.6)	11 (7.3)	28 (10.8)
Home	33 (30.3)	30 (20.0)	63 (24.4)
On route to hospital or facility	2 (1.8)	3 (2.0)	5 (1.9)
**Cause of death**			
Prematurity	58 (53.2)	55 (36.7)	113 (43.6)
IPRE (mainly birth asphyxia)	36 (33.0)	64 (42.7)	100 (38.6)
Neonatal sepsis	7 (6.5)	17 (11.2)	24 (9.2)
Neonatal pneumonia	5 (4.6)	10 (6.7)	15 (5.8)
Other causes ^#^	3 (2.7)	4 (2.7)	7 (2.8)

^+^ Of 37 neonates delivered at home, the birth was attended by midwives (14%), TBA (32%), relative/friend (8%), no one (46%—due to precipitous delivery)

^#^Other causes of death: congenital malformation, accidental fall, road traffic accident, and meningitis and encephalitis

### Cause of deaths

**Fig 1:**
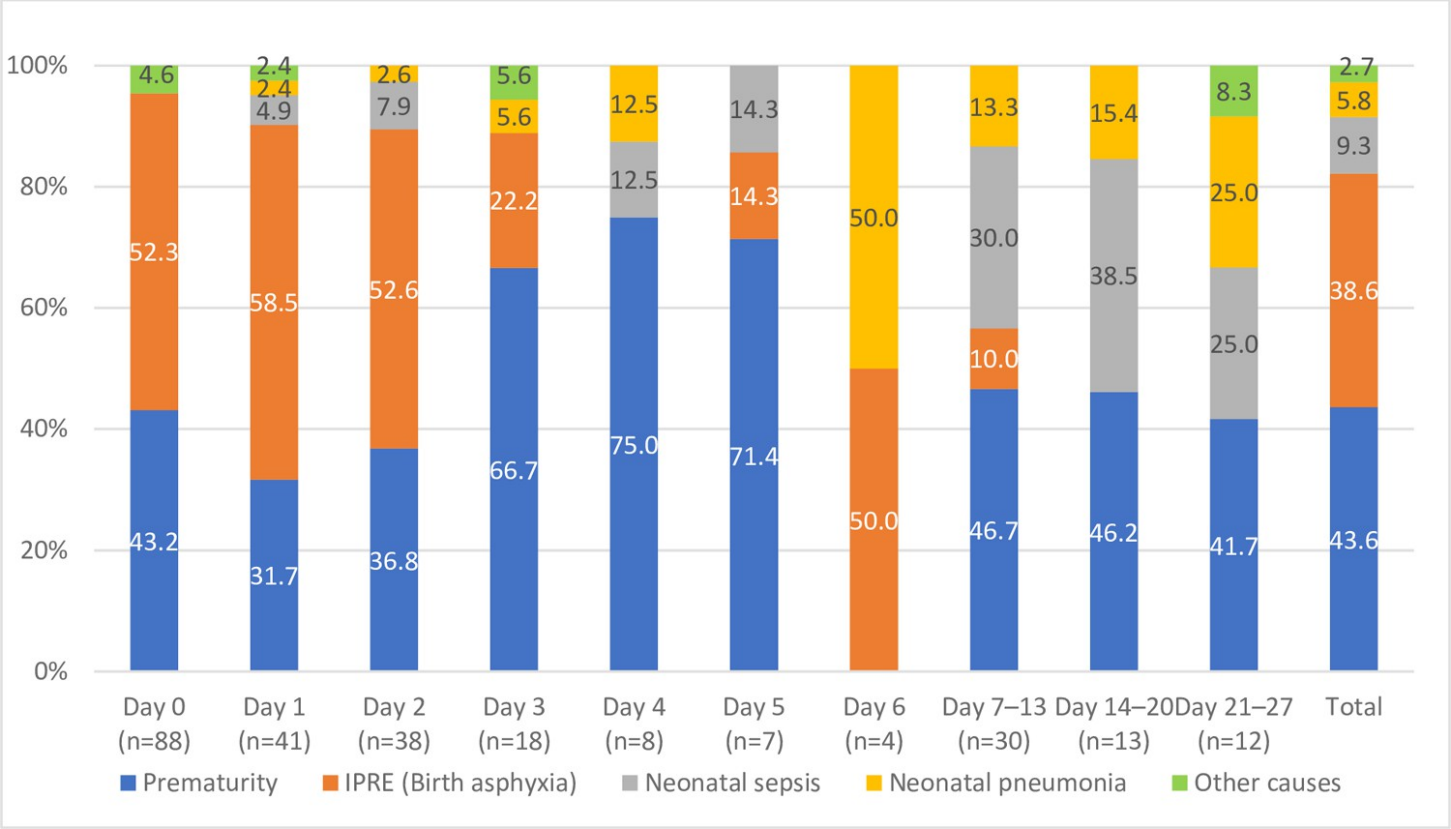
Cause of death by postnatal age.

Table 3 shows that prematurity, intrapartum-related events (IPRE)- mainly birth asphyxia, and neonatal sepsis were the three leading causes of death, accounting for 92% of all neonatal deaths (44%, 39%, and 9% of deaths, respectively). Fig 1 shows the distribution of age at death and the causes of death; it reveals that a high proportion of deaths due to IPRE in the first week occurred during days 0–2 and the proportion of deaths due to prematurity gradually increased after day two. Prematurity was the major cause of death in weeks two to four.

### The care-seeking pathway

**Fig 2:**
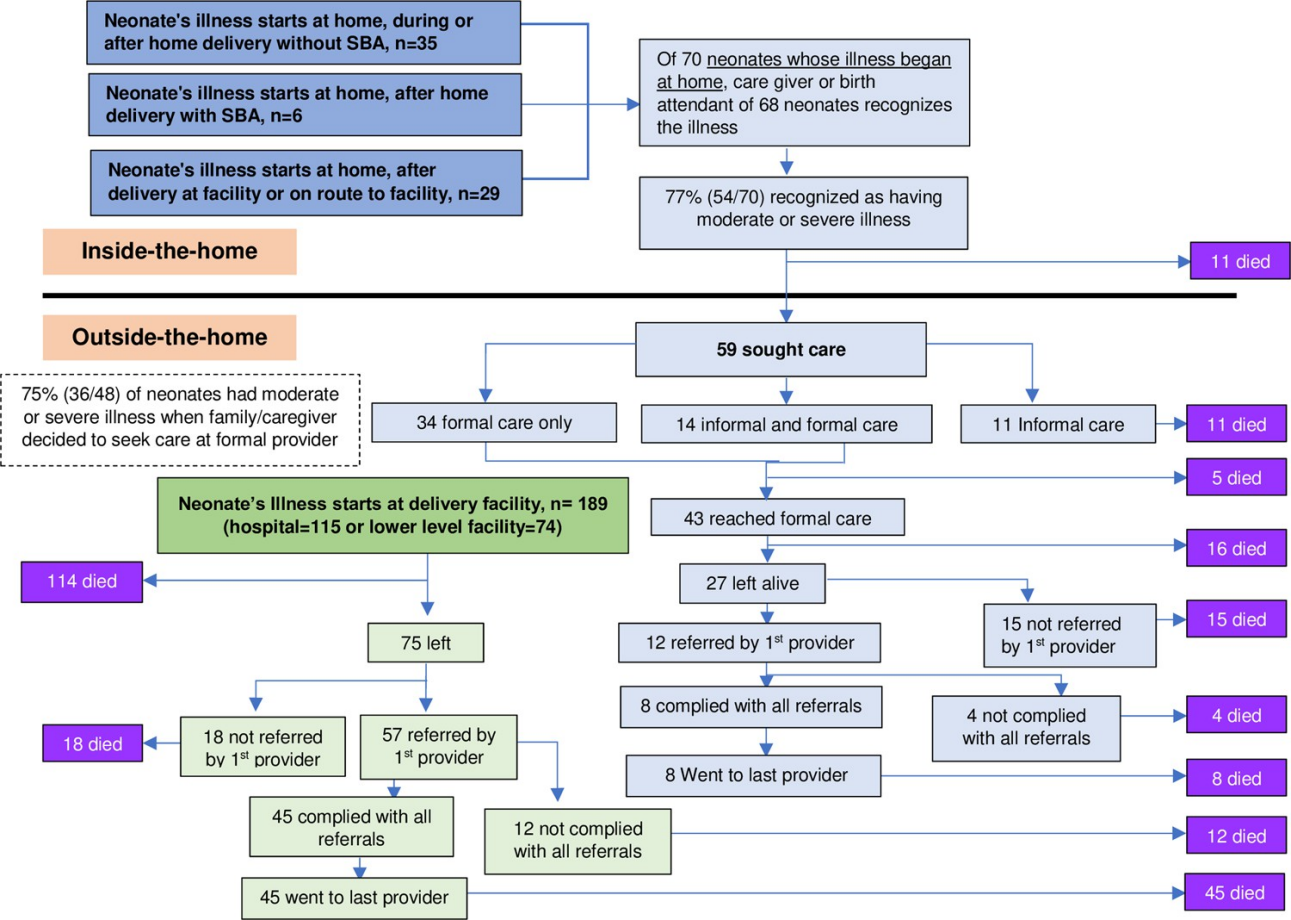
Care-seeking pathway.

Fig 2 shows the care-seeking pathway of neonates based on place where the fatal illness started. Steps in the pathway are aligned with the results presented in Table 4. This table shows the same care-seeking pathway of neonates, separately for those whose fatal illness began at home and at facilities where the neonates were delivered.

**Table 4:** Steps in care seeking pathway.

Pathway Steps	Neonates whose fatal illness began at home (n = 70)			Neonates whose fatal illness began at delivery facility (n = 189)	
	Neonate’s illness starts during or after home delivery without SBA (n = 35) n (%)	Neonate’s illness starts after home delivery with SBA (n = 6) n (%)	Neonate’s illness starts after delivery at a health facility or on route to a health facility (n = 29) n (%)	Neonate’s illness starts at a lower level facility (n = 74) n (%)	Neonate’s illness starts at hospital (n = 115) n (%)
**Died before seeking outside care**	**7/35 (20)**	**0/6 (0)**	**4/29 (14)**		
**Sought care**	**28/35 (80)**	**6/6 (100)**	**25/29 (86)**		
Formal provider only	10/28 (36)	6/6 (100)	18/25 (72)		
Informal and formal providers	10/28 (36)	0/6 (0)	4/25 (16)		
Informal provider only**	8/28 (28)	0/6 (0)	3/25 (12)		
**Severity when deciding to seek care at formal care provider**					
Normal	1/20 (5)	0/6 (0)	5/22 (23)		
Mild	2/20 (10)	0/6 (0)	2/22 (9)		
Moderate	8/20 (40)	0/6 (0)	6/22 (27)		
Severe	9/20 (45)	5/6 (83)	8/22 (36)		
DK	0/20 (0)	1/6 (17)	1/22 (5)		
**Reached 1**^**st**^ **formal care provider alive**	17/20 (85)	6/6 (100)	20/22 (91)	74/74 (100)	115/115 (100)
**Use of insurance at 1**^**st**^ **formal care provider**					
Insured	2/20 (10)	1/6 (17)	3/22 (14)	16/74 (22)	47/115 (41)
Government aid	1/20 (5)	3/6 (50)	0/22 (0)	14/74 (19)	28/115 (24)
No insurance	17/20 (85)	2/6 (33)	19/22 (86)	44/74 (59)	40/115 (35)
**Left 1**^**st**^ **formal care provider alive**	13/17 (76)	0/6 (0)	14/20 (70)	51/74 (69)	24/115 (21)
**Severity when leaving 1**^**st**^ **formal provider**					
Normal	1/13 (8)		4/14 (29)	1/51 (2)	1/24 (4)
Mild	2/13 (16)		1/14 (7)	4/51 (8)	3/24 (12)
Moderate	5/13 (38)		5/14 (35)	16/51 (31)	4/24 (17)
Severe	5/13 (38)		4/14 (29)	28/51 (55)	15/24 (63)
DK	0/13 (0)		0/14 (0)	2/51 (4)	1/24 (4)
**Referred by 1**^**st**^ **formal care provider**	7/13 (54)		5/14 (36)	41/51 (80)	16/24 (67)
To lower level health facilities	4/7 (57)		2/5 (40)	4/41 (10)	0/24 (0)
To hospital	3/7 (43)		3/5 (60)	37/41 (90)	15/16 (94)
DK	0/7 (0)		0/5 (0)	0/41 (0)	1/16 (6)
**Complied with all referrals**	3/7 (43)		5/5 (100)	34/41 (83)	11/16 (69)
Went to a last provider	3/3 (100)		5/5 (100)	34/34 (100)	11/11 (100)

SBA = skilled birth attendant; Grey highlighted sections indicate not applicable (NA); lower level facility means primary level facilities (below hospital) including health centres, midwifery practices, maternity clinics, etc.

The 70 neonates whose fatal illness started at home includes those who were delivered at home without an SBA (n = 35), neonates whose fatal illness started at home after home delivery with an SBA (n = 6), and neonates whose fatal illness started at home after being born at a health facility or on route to a health facility (n = 29). Seventy-seven percent (54/70) of the neonates in this group were moderately or severely ill when the family recognized the illness (not shown in Table 4), and 11 died before any care was sought. Of the 59 for whom care was sought, only 81% (n = 48) went to formal care providers (either formal only or a combination of formal and informal care) and 75% (n = 36) of these 48 neonates were moderately or severely ill when the family decided to seek care. Of the 11 neonates taken for informal care only, the main reasons for not seeking formal care were: distance to provider (19%), ‘no hope’ for the neonate’s illness (19%), and unavailability of either transportation or a formal provider (19%).

For the first formal care received, the parents of 79% (38/48) of neonates did not use any insurance. After receiving care, 63% (n = 27) of 43 neonates who reached a first provider were discharged alive. However, 70% (19/27) of those who left the first provider alive were in moderate to severe condition upon discharge. Ten of those 19 neonates were referred to another health facility. Two other neonates were not in a moderate or severe condition when leaving the first provider and were also referred.

The other group shown in Table 4 consists of 189 neonates whose fatal illness began at the delivery facility, either at a hospital or a lower level facility. Having no insurance was less common among neonates who were delivered at a hospital (35%; 40/115) compared to those who were born at a lower health facility (59%; 44/74). Of the 115 neonates who were born at a hospital, only 21% left the hospital alive, compared to 69% of those born at a lower level facility.

Most neonates whose fatal illness began at the delivery facility and left alive were in moderate or severe conditions upon discharge. Neonates whose illness started at the delivery facility were more likely to be referred to another facility (76%; 57/75) than those whose illness started at home and were then taken to a health facility (44%; 12/27). Of the 57 referred neonates whose fatal illness began in their delivery facility and left alive, 52 (91%) were referred to a hospital, compared to 6 of the 12 neonates (50%) whose illness began at home were referred by their first provider. Sixty-nine percent of neonates referred by a hospital and 83% of those referred to a lower health facility complied with the referral recommendation.

## Discussion

There are few population-based studies that have examined neonatal mortality at a sub-national level in Indonesia. In this paper, we highlight issues that focus on care-seeking during fatal illness and the causes of neonatal death. Findings from this study can assist decision makers at the district level to devise strategies to reduce neonatal mortality with a focus on modifiable neonatal care factors.

Most families of neonates whose fatal illness started at home sought care. However, illness recognition and formal health care-seeking were delayed. About one-sixth of the neonates died before any care was sought, another sixth went to informal instead of formal care providers, and three-quarters of those for whom formal care was sought were moderately or severely ill before the decision was made to seek care. While in our study a small percentage of neonates went only to informal care providers, mainly due to physical constraints and resource limitations, other studies have shown that lack of knowledge of danger signs of neonatal illness and traditional beliefs may play a role in the choice of care provider [30].

Many infants were born and died in the same facility, while others were discharged and referred while in still a moderately to severely ill condition. This may indicate that the neonates needed a higher level of care than could be provided at the place of birth. Furthermore, there was a high proportion of neonates whose fatal illness started at the hospital where they were born and who were then referred to another hospital. This may suggest issues with hospital readiness to provide care, staff’s capacity to provide care, or that the cost for care was beyond the family’s ability to pay.

The issue of referral is important and needs closer examination in the Indonesian context. For example, a case-control study in Brazil indicated that unnecessary transfer of newborns to other facilities after birth may increase the risk of death due to poor quality of care at the places of referral [31]. Future research is also needed to evaluate the quality of neonatal care provided, given the fact that most of the deceased neonates had reached a health facility alive.

Elsewhere, NHI has been shown to improve utilization of maternal health services, including in Indonesia [8, 32]. The association between insurance and care-seeking for neonatal illness, however, remains inconclusive. One study in Taiwan found no significant impact of NHI on the use of neonatal preventive care and vaccination, although NHI did lessen the inequality of these two services’ utilization in some regions [33].

In our study, the families of most of the deceased neonates did not use insurance when they received care at the first point of contact even if they had it; about one-third of the families used insurance, and another 18% received government aid. Among neonates who were born and became ill at a hospital, the high proportion of those who used insurance or government aid may point to two dynamics. First, the mothers may have had severe maternal complications that required them to deliver at hospital, and subsequently the neonates developed illness during hospitalization. With emergency situations needing hospital care, it is common in Indonesia that government aid is offered to those who have no insurance and poor. Second, the mothers who neither had insurance nor received government aid may have preferred to give birth at lower-level facilities.

Our study showed that the proportion of neonates without any insurance at the first point of care for their illness (53%) was higher than for those who did not have insurance for their maternal care (34%). The fact that some women had insurance for maternal care but their neonates did not, suggests that either: community awareness of the need to register their babies’ births and for insurance coverage immediately after birth was low; or there was a lack of understanding about the procedure for registering their neonates, especially when the neonates had fatal illness soon after birth [13].

During the interviews, some respondents expressed difficulties in receiving insurance or government aid for their neonates, which required 14 days waiting time at the time of this study. This is perhaps due to the neonate not yet having been added to the family registration card. This card is used as the basis for registering for insurance or government aid. It should be noted, however, that the government made changes to the NHI regulations in late 2018, after the end of our study period. According to the new regulation, registration of NHI membership for neonates can be completed within 28 days after birth for non-subsidized NHI members without a waiting period, and further, that neonates of women who received subsidized NHI would be automatically covered [34]. The new regulation of the NHI for neonates [34] deserves evaluation to understand the implementation and its effect on neonatal outcomes.

Consistent with findings from other countries, preterm birth, IPRE, and infection were the main causes of neonatal death [1]. However, we found a higher proportion of deaths due to prematurity (44%) compared to the global estimate (35%) [1]. Similar to preterm birth, our data also show a higher percentage of deaths due to IPRE than the global estimate, 39% versus 24% [1]. Studies have shown that IPRE is more closely associated with maternal complications and early neonatal care that can be improved through interventions such as improved labor and delivery management and immediate neonatal resuscitation [35–37].

Survival of premature and sick neonates may be increased by better neonatal care such as implementing a simple intervention like kangaroo mother care (KMC), providing competent resuscitation, and increasing access to neonatal intensive care units (NICU). Each study district had a limited number of NICUs available, as is the case for most districts in Indonesia.

### Study limitations

This study analyzed data from neonatal deaths without a control group, and thus, we could not analyze risk factors for neonatal mortality. The care-seeking pattern described in the study may not represent neonates in general. The fact that care was sought for nearly all deceased neonates, but mostly when the illness was moderate or severe, deserves in-depth examination about the dynamics of care-seeking and the provision of high-quality care for sick neonates. However, this study was not designed to assess quality of care, thus we cannot describe the quality of care once a patient reached a health facility. Lastly, we attempted to depict the distribution of age at death by cause of death in Fig 1, however, due to the small sample of cases from days 3 to 5, interpretation of the results should be made with caution.

## Conclusions

This study highlights several areas of potential interventions to improve outcomes for neonates in Jember and Serang Districts of Indonesia. First, the high proportion of deaths due to prematurity and IPRE suggests the need for better care for small and sick neonates such as by implementing KMC, competent resuscitation, and availability of NICUs. Second, a high percentage of families sought care for neonates who were in moderate to severe condition suggests the importance of awareness about illness recognition and care-seeking to improve the health and survival of neonates in cases where the neonates are not already in a health facility. Finally, among neonates whose illness began in the delivery facility, the high proportion of neonates recommended for referral and the severity of the neonates’ condition when leaving the facility raises concerns about the quality, capacity, and/or cost of care at delivery facilities.
